# Delayed sternal closure does not reduce complications associated with coagulopathy and right ventricular failure after left ventricular assist device implantation

**DOI:** 10.1007/s10047-017-0996-z

**Published:** 2017-09-25

**Authors:** Roh Yanagida, Navin Rajagopalan, Daniel L. Davenport, Thomas A. Tribble, Mark A. Bradley, Charles W. Hoopes

**Affiliations:** 1 Department of Cardiothoracic Surgery, Newark Beth Israel Medical Center, 201 Lyons Avenue, Newark, NJ 07112 USA; 2 Division of Cardiology, University of Kentucky, Lexington, USA; 3 Department of Surgery, University of Kentucky, Lexington, USA; 4 Division of Cardiothoracic Surgery, Department of Surgery, University of Kentucky, Lexington, USA; 5 Division of Cardiothoracic Surgery, Department of Surgery, University of Alabama, Birmingham, USA

**Keywords:** Left ventricular assist device, Delayed sternal closure, Right ventricular failure, Vacuum assist device, Coagulopathy

## Abstract

Delayed sternal closure (DSC) is occasionally adopted after implantation of left ventricular assist device (LVAD). Recent studies suggest that DSC be used for high risk group of patients with coagulopathy, hemodynamic instability or right ventricular failure. However, whether DSC is efficacious for bleeding complication or right ventricular failure is not known. This study is single center analysis of 52 patients, who underwent LVAD implantation. Of those 52 patients, 40 consecutive patients underwent DSC routinely. The sternum was left open with vacuum assist device after implantation of LVAD. Perioperative outcome of the patients who underwent routine DSC were compared with 12 patients who had immediate sternal closure (IC). Mean Interagency Registry for Mechanically Assisted Circulatory Support (INTERMACS) level of IC group and DSC group were 2.7 and 2.6, respectively. Postoperative bleeding (643 vs. 1469 ml, *p* < 0.001), duration of inotropic support (109 vs. 172 h, *p* = 0.034), and time to extubation (26 vs. 52 h, *p* = 0.005) were significantly increased in DSC group. Length of ICU stay (14 vs. 15 days, *p* = 0.234) and hospital stay (28 vs. 20 days, *p* = 0.145) were similar. Incidence of right ventricular failure and tamponade were similar in the two groups. Routine DSC after implantation of an LVAD did not prove to be beneficial in reducing complications associated with coagulopathy and hemodynamic instability including cardiac tamponade or right ventricular failure. We suggest that DSC be selectively applied for patients undergoing LVAD implant.

## Introduction

Bleeding after implantation of a left ventricular assist device (LVAD) is common. Reported incidence of reexploration for bleeding in this patient population is approximately 30% [1, 21, 2]. For diffuse bleeding in the mediastinum after surgical hemostasis, the sternum may be left open. Delayed sternal closure (DSC) may be occasionally adopted for other reasons such as hemodynamic instability, which may be associated with right ventricular failure in this group of patients with chronic heart failure. However, its indications have not been clear.

In a recent retrospective study that included 184 patients who underwent DSC after LVAD implant, DSC was utilized most commonly for coagulopathy, hemodynamic instability and prior sternotomy. DSC was associated with longer ICU stay but it was not associated with a significantly increased risk of death or infection [3]. In another study that analyzed this patient population, DSC was adopted for 16.8% of those undergoing LVAD with high risk characteristics including higher use of intraoperative blood products, longer cardiopulmonary bypass time and right ventricular assist device (RVAD) support [4]. DSC may be helpful to reduce morbidity and mortality of these high risk patients. However, efficacy of DSC, whether DSC reduces bleeding complication or right ventricular failure, is not known.

In this study, we made a hypothesis that DSC reduces complications associated with coagulopathy and hemodynamic instability, such as cardiac tamponade or right ventricular failure, for patients undergoing LVAD implantation. To determine this hypothesis, we performed DSC with a wound vacuum device routinely after LVAD implant and closed the chest the following day. The occurrence of bleeding complications, cardiac tamponade, or right ventricular failure was retrospectively reviewed. The patients with DSC were compared with those with immediate closure (IC).

## Methods

From May 2008 to October 2012, 52 patients underwent insertion of HeartMate II LVAD (Thoratec Corp., Pleasanton, CA) at our institution. First 12 patients had the chest closed at the initial operation (IC group, *n* = 12). Thereafter, 40 patients underwent routine DSC with wound vacuum device (DSC group, *n* = 40). All patients enrolled in this study have given their informed consent, which has been approved by the institutional committee and this protocol has been found acceptable by them.

DSC was performed as the following. After insertion of an LVAD, hemostasis was done and 32 Fr. chest tubes were placed in the mediastinum inferior and anterior to the ventricle. The chest was temporarily closed with wound vacuum device (Kinetic Concepts Inc., San Antonio, TX). A WhiteFoam^®^ dressing was placed on the anterior wall of the right ventricle. A black Simplace^®^ dressing was placed above the WhiteFoam^®^ dressing to fill the space between the sternal edge and the skin level. A thin plastic drape was placed on the chest and continuous vacuum was started at 25–50 mmHg depending on the amount of bleeding and chest tube output [5]. Chest tubes were placed on suction at 20 cmH_2_O.

Postoperatively continuous intravenous infusion of propofol (0.01–0.05 mg/kg/min) was used to lightly sedate the patient after recovery of consciousness and gross neurologic examination. Inotropes and vasopressors regimen included epinephrine, norepinephrine and milrinone, and were titrated to maintain mean arterial pressure above 60 mmHg and adequate urine output. Nitric oxide, started at 20 parts per million, was used when there were signs of right ventricular dysfunction including elevation of central venous pressure greater than 15 mmHg or echocardiographic finding of right ventricular dysfunction. On postoperative day 1 the chest was closed in the operating room in the standard fashion. All the mediastinal clots were removed. The patients were mobilized from the bed several hours after extubation. Postoperative antibiotics included vancomycin and rifampin and piperacillin/tazobactam for 3 days. For patients with penicillin allergy, cefepime instead of piperacillin/tazobactam was given for 3 days.

Continuous variables were summarized by mean ± standard deviation. Normally distributed continuous variables were compared across two groups by the independent samples *t* test. Non-normally distributed variables were compared across two groups by the Wilcoxon rank sum test. Categorical variables were summarized by frequency and percent. Categorical variables were compared across groups by the Fisher’s exact test. A *p* value < 0.05 was considered significant. SPSS™ statistical software version 20 (IBM Corporation, New York, NY) was used for statistical analysis.

## Results

Baseline clinical characteristics of the study patients are shown in Table 1. INTERMACS profile level was similar in two groups. Twelve patients (25%) were supported by mechanical circulatory support before an LVAD insertion in the study patients, 2 in IC and 10 in DSC. Pre-LVAD mechanical circulatory support included veno-arterial extracorporeal membranous oxygenation for 3 patients, intra-aortic balloon pump (IABP) for 3, HeartMate I (Thoratec Corp.) for 1, biventricular support with CentriMag (Thoratec Corp.) for 1, CentriMag LVAD with IABP for 1, CentriMag right ventricular assist device (RVAD) with IABP for 1, CentriMag RVAD for 1 and Impella 5.0 (Abiomed, Danvers, MA) for 1.

**Table 1:** Baseline clinical characteristics

	All (*n* = 52)	IC (*n* = 12)	DSC (*n* = 40)	*p*
Age (years)	54.4 ± 12.7	59.4 ± 11.5	52.9 ± 12.8	0.119
Female	14 (27%)	2 (17%)	12 (30%)	0.475
BSA (m^2^)	2.1 ± 0.3	2.2 ± 0.3	2.1 ± 0.2	0.407
BMI (kg/m^2^)	30.1 ± 6.5	32.2 ± 7.4	30.3 ± 6.3	0.385
Mean INTERMACS level	3	2.7	2.6	N/A
Previous sternotomy	15 (29%)	2 (18%)	13 (33%)	0.472
Bridging device to LVAD	12 (23%)	2 (18%)	10 (25%)	0.706
Mechanical ventilation	7 (13.5%)	1 (9.1%)	6 (15%)	1.000
Inotropes	21 (41%)	6 (55%)	15 (38%)	0.327
Ischemic cardiomyopathy	36 (69%)	6 (50%)	30 (75%)	0.153
DT (vs. BTT)	23 (44%)	4 (33%)	19 (48%)	0.153

*BMI* body mass index, *BSA* body surface area, *BTT* bridge to transplant, *DSC* delayed sternal closure group, *DT* destination therapy, *IC* immediate sternal closure group, *INTERMACS* Interagency Registry for Mechanically Assisted Circulatory Support, *LVAD* left ventricular assist device

Preoperative laboratory data and hemodynamics are shown in Tables 2 and 3, respectively. No significant difference was identified between the two groups.

**Table 2:** Preoperative laboratory data

	All (*n* = 52)	IC (*n* = 12)	DSC (*n* = 40)	*p*
Serum Cr (mg/dL)	1.5 ± 0.6	1.7 ± 0.8	1.4 ± 0.5	0.132
Total Bil. (mg/dL)	2.1 ± 2.9	1.4 ± 1.3	2.3 ± 3.2	0.385
AST (U/L)	134 ± 486	32 ± 23	162 ± 547	0.438
Albumin (g/dL)	3.1 ± 0.6	3.0 ± 0.7	3.1 ± 0.5	0.524
Hb (g/dL)	11.3 ± 2.5	10.6 ± 1.9	11.4 ± 2.6	0.302
Plt (k/μL)	197 ± 113	230 ± 194	188 ± 79	0.502
INR	1.8 ± 1.1	1.8 ± 0.7	1.7 ± 1.2	0.964

*AST* aspartate aminotransferase, *Bil* bilirubin, *Cr* creatinine, *DSC* delayed sternal closure group, *Hb* hemoglobin, *IC* immediate sternal closure group, *INR* international normalized ratio, *Plt* platelet

**Table 3:** Preoperative hemodynamics

	All (*n* = 52)	IC (*n* = 12)	DSC (*n* = 40)	*p*
HR (/min)	86 ± 22	83 ± 29	92 ± 21	0.274
Systolic BP (mmHg)	100 ± 19	96 ± 13	102 ± 18	0.290
Diastolic BP (mmHg)	59 ± 15	63 ± 14	56 ± 11	0.147
CI (l/min/m^2^)	2.0 ± 0.4	2.1 ± 0.6	1.8 ± 0.3	0.052
CVP (mmHg)	19 ± 7	19 ± 6	19 ± 7	0.892
PCWP (mmHg)	30 ± 8	29 ± 8	30 ± 7	0.793
mPAP (mmHg)	43 ± 12	44 ± 11	43 ± 12	0.793

*BP* blood pressure, *CI* cardiac index, *CVP* central venous pressure, *HR* heart rate, *mAP* mean arterial pressure, *mPAP* mean pulmonary arterial pressure, *PCWP* pulmonary capillary wedge pressure

Postoperative outcome is summarized in Table 4. Operation time was similar in the two groups. Concomitant surgery was performed in 10 patients (19%), 3 (25%) in IC and 7 (18%) in DSC, including 4 RVADs, 4 tricuspid valve repairs, 3 aortic valve replacements, 1 atrial septal defect and 1 patent foramen ovale closure. Use of factor VII was more common in DSC (*p* = 0.02). Postoperative bleeding, duration of inotropic support and time to extubation were significantly increased in DSC. Length of ICU stay and hospital stay were similar in the two groups. There was no sternal infection in either group. 30 day and 1 year survival were similar in the two groups.

**Table 4:** Postoperative clinical characteristics

	All (*n* = 52)	IC (*n* = 12)	DSC (*n* = 40)	*p*
OR time (min)	321 ± 115	332 ± 135	318 ± 110	0.753
CPB time (min)	111 ± 51	92 ± 22	115 ± 55	0.322
Concomitant surgery	10 (19%)	3 (25%)	7 (18%)	0.669
Use of factor VII	16 (31%)	0	16 (41%)	0.020
Postoperative bleeding in the first 24 h (ml)	1278 ± 944	643 ± 300	1469 ± 989	< 0.001
Postoperative PRBC (unit)	3.3 ± 1.2	3.3 ± 1.0	3.2 ± 1.3	0.564
Reexploration for bleeding	2 (4%)	1 (8%)	1 (3%)	0.412
Cardiac tamponade	3 (6%)	1 (8%)	2 (5%)	0.553
RV failure	6 (12%)	0	6 (15%)	0.316
Sternal infection	0	0	0	N/A
Driveline infection	5 (10%)	2 (17%)	3 (8%)	0.325
Inotropic support (h)	158 ± 90	109 ± 70	172 ± 91	0.034
Time to extubation (h)	46 ± 42	26 ± 23	52 ± 45	0.005
ICU stay (days)	15 ± 16	14 ± 8	15 ± 18	0.234
LOS (days)	22 ± 17	28 ± 19	20 ± 17	0.145
30 day survival (%)	90	92	90	1.000
1 year survival (%)	74	83	70	0.693

*CPB* cardiopulmonary bypass, *DSC* delayed sternal closure group, *IC* immediate sternal closure group, *ICU* intensive care unit, *LOS* length of stay, *OR* operating room, *PRBC* packed red blood cell, *RV* right ventricle

Total direct cost per case excluding provider tax is shown in Table 5. The cost was similar in the two groups. Costs for surgery and anesthesia were greater in DSC but nursing ICU cost was lower in DSC.

**Table 5:** Total cost per case for implantation of LVAD (excluding provider tax)

	IC	DSC
Period (year)	2008–2010	2011–2012
Total ($, range)	195,171 (189,142–204,053)	188,644 (182,357–194,430)
Surgery ($, range)	107,706 (96,294–121,002)	131,601 (128,086–135,117)
Anesthesia ($, range)	4155 (3338–4608)	4249 (3692–4085)
Nursing ICU ($, range)	33,155 (27,300–41,842)	17,749 (14,033–21,465)
Pharmacy ($, range)	13,556 (10,171–19,807)	8679 (6719–10,639)

*DSC* delayed sternal closure group, *IC* immediate sternal closure group, *ICU* intensive care unit, *$* dollars

## Discussion

The present study reviewed outcome of patients who underwent DSC after implantation of LVAD. DSC did not reduce incidence of bleeding complications, cardiac tamponade or right ventricular failure compared to IC. DSC increased postoperative bleeding, time to extubation but not length of stay in ICU. Objective of our study was different from other retrospective studies in which DSC were selectively applied. Contrary to those studies which identified risk factors that required DSC, our study attempted to determine efficacy of DSC.

The sternum may be left open after implantation of LVAD [2–4]. DSC may be adopted for reasons including bleeding, hemodynamic instability, right ventricular failure and arrhythmia. When it is done for bleeding, theoretical benefit may include less occurrence of cardiac tamponade by providing more pericardial space. It also provides benefit of immediate access for mediastinal exploration if needed in the ICU for acute change in hemodynamics. Bleeding in patients with DSC was approximately 820 ml greater than in patients with IC in the first 24 h in the present study. Persistent bleeding was frequently noticed from the vacuum assist device while there was minimal bleeding from the mediastinal chest tubes. Vacuum assist device was occasionally sucking blood from the bone marrow of the sternum since the negative pressure applied by the vacuum assist device was greater than negative pressure applied by 32 Fr. chest tubes in the mediastinum. In a case study that reported use of wound vacuum assisted closure to control coagulopathic bleeding in a patient after insertion of LVAD, it was theorized that a tamponade effect between the mediastinal tissue and surgical sponges is established by the negative pressure applied by the chest tubes placed in pleural cavities bilaterally [6]. In our experience, negative pressure could not be applied effectively on the entire mediastinum due to communication with chest tubes and blood loss appeared to be greater due to bleeding from the sternum. Amount of blood transfusion was similar in both groups. This may be explained by our practice of restrictive strategy and minimizing amount of blood transfusion to prevent immunomodulation effect for potential future transplant.

We did not observe significant difference in the incidence of right ventricular failure in two groups of patients regardless of the sternum was left open or closed at the time of LVAD implantation; none in IC and 6 patients (15%) in DSC (*p* = 0.316). Sternal closure has been shown to result in a significant decrease in cardiac output and diastolic filling even in patients with good cardiac performance [7]. Furnary et al. has demonstrated that low cardiac output can be improved by the opening of the sternum [8]. With this evidence one may think that leaving the chest open would help prevent right ventricular failure potentially precipitated by sternal closure. Right ventricular failure can occur at any time after insertion of LVAD. Most of RVAD insertion after LVAD implant occurs in the operating room at the time of LVAD insertion or within 24 h [9]. In our group of patients with DSC it was possible that right ventricle, which would have developed right ventricular failure recovered in the first 24 h while the chest was open. However, owing to the retrospective nature of the study, our results do not provide more insight into this issue.

No patients had sternal infection regardless of timing of chest closure in the current study. DSC as a risk factor for sternal infection has been debated [10, 11]. In a recent study that included 5177 patients who underwent cardiac procedures, 87 patients (1.7%) were managed with DSC. Incidence of sternal infection was 4.6%. Redo operations, insertion of VAD, tracheostomy and prolonged open chest were potential risk factors for sternal infection [11]. On the other hand, more recently in patients after LVAD implantation, Stulak et al. reported that there was no increase in the incidence of infectious complication in 184 patients who had DSC after LVAD implantation [3]. Results in our study support this finding. Although further studies with more patients are needed to conclude, DSC can be safely applied for patients implanted with a VAD without increasing sternal infectious complications.

In the present study, DSC prolonged time to extubation since the patients stayed intubated until the chest was closed the following day. For the same reason DSC prolonged the use of IV inotropes since weaning of inotropes usually started after the sternum was closed. It was expected that this would lead to longer ICU stay in patients after DSC; however, there was no difference between the two groups. To minimize ICU stay early extubation and ambulation were encouraged. Early ambulation, which is 4 to 6 h after extubation, was still possible in patients after DSC. There was no difference in overall cost for hospital admission in two groups. It was attributed to early mobilization in the postoperative management of patients after DSC. At the same time management of LVAD patients in the ICU became more efficient as more VAD implantations were performed.

Our study did not prove routine DSC to be advantageous and suggests that DSC be selectively applied. Question remains in terms of which patients should have DSC after implantation of LVAD. There are patients who continue to bleed after insertion of LVAD, especially in patients with previous sternotomy or on platelet inhibitors. These bleeding patients with cardiomyopathy are susceptible to hemodynamic instability and threshold to reexploration should be low. Although DSC was not found useful to decrease the incidence of cardiac tamponade or right ventricular failure in our experience, there is still an added advantage of allowing immediate access to the mediastinum for evacuation of blood and clots for cardiac tamponade and for application of internal defibrillation if needed in ICU. In studies that analyzed patients in whom DSC was adopted after LVAD insertion, indication was mainly for coagulopathy. Two recent studies described use of DSC in this patient population [3, 4]. In Stulak’s study, reasons for DSC included coagulopathy in 155 patients (84%), hemodynamic instability in 103 (56%), significant isolated right ventricular dysfunction in 15 (8%). The other study that analyzed use of DSC after LVAD implant did not mention reason for DSC but identified predictors of DSC including preoperative glycoprotein IIb/IIIa inhibitor, more intraoperative use of PRBC and tricuspid valve procedure [4]. For coagulopathic patients DSC actually increases bleeding but facilitates subsequent reexploration. For patients with tenuous right ventricular function DSC will be needed if the patient does not tolerate sternal approximation, however, DSC itself will not reduce incidence of postoperative right ventricular dysfunction.

### Limitations

Our case series is limited by a small number of patients and retrospective assessment of data and it is subject to all limitations inherent in such studies. In this observational study, sample sizes of patients with DSC and IC were not balanced. This sample size imbalance was partly due to increasing case volume during the study period as the number of referrals to our mechanical circulatory support program increased. DSC was introduced to our practice as the program was increasing case volume. As case volume increased the patients who needed LVAD became more heterogeneous, although two groups are statistically similar in collected variables. Lack of balance in sample size may make finding a significant difference more difficult. Failure to detect a difference may be a result of low power due to small sample size. Brain natriuretic peptide measurement was not routinely done early in the study period and was not shown in this study. Echocardiographic finding was not shown in this study since diagnosis of right ventricular failure after VAD implantation was made clinically including hemodynamic data. From our visual observation, increase in blood loss in DSC was attributed to continuous marrow bleed from the sternum; however, blood loss was measured as a combined output from the wound vacuum device and the chest tubes. Separate measurement of two drainage sources was not performed in this study. To more strictly compare the amount of bleeding, all the blood products given for bleeding should be measured. In our study, only the amount of packed red blood cell and factor VII were included as parameters.

## Conclusions

Routine DSC after implantation of an LVAD did not prove to be beneficial in reducing complications associated with coagulopathy and hemodynamic instability including cardiac tamponade or right ventricular failure. Although it increased postoperative bleeding and time to extubation, it did not increase hospital length of stay, incidence of sternal infection or driveline infection. We suggest that DSC be selectively applied for patients undergoing LVAD implant.
